# Focus on High School: Factors Associated with Creating Harmony between the Educational Transition and Adolescents’ Well-Being

**DOI:** 10.3390/ijerph19159261

**Published:** 2022-07-28

**Authors:** Pheerasak Assavanopakun, Wachiranun Sirikul, Tharntip Promkutkao, Suchat Promkutkeo, Jinjuta Panumasvivat

**Affiliations:** 1 Department of Community Medicine, Faculty of Medicine, Chiang Mai University, Chiang Mai 50200, Thailand; pheerasak.assava@cmu.ac.th (P.A.); tharntip.p@cmu.ac.th (T.P.); jinjuta.p@cmu.ac.th (J.P.); 2 Center of Data Analytics and Knowledge Synthesis for Health Care, Chiang Mai University, Chiang Mai 50200, Thailand; 3 School of Economics, Chiang Mai University, Chiang Mai 50200, Thailand; suchat.promkutkeo@cmu.ac.th

**Keywords:** high school, adolescents’ well-being, school reopening, educational transition

## Abstract

The transition from online to on-site education was difficult due to a lack of standardized school guidance for school reopening. Even though schools have reopened, uncertainty about the COVID-19 situation and the capacity of the school to maintain safe school operations to mitigate risks may increase hesitancy among students and parents to participate in on-site studying. Rapid-response surveys of students and parents can provide information to stakeholders on how learning and well-being can best be supported during the educational transition in each context. The aim of this study was to explore the hesitancy of high-school students and the factors that influenced their hesitancy to return to school on site. An online cross-sectional survey was distributed to high-school students in an urban district of Chiang Mai, Thailand, during the fourth wave of the pandemic from 17 November to 13 December 2021. A multivariable logistic regression was performed to explore factors related to the students’ hesitancy to attend on-site education. With a response rate of 10.2% of the targeted study population, the 1266 participants revealed that 15.9% of them had very-high- and extremely high-level hesitancy to study on site, which was associated with less negative moods while studying online (aOR, 1.69; *p*, 0.016) and a greater fear of infection after returning to school (aOR, 2.95; *p*, 0.001). Increased readiness to return to school on site (aOR, 0.28; *p*, 0.001) and discussing COVID-19 prevention with family or friends (aOR, 0.71; *p*, 0.016) were also associated with a lower hesitancy of students. Only 5.6% of the students reported being hesitant to receive the COVID-19 vaccination. However, no significant associations were found between schooling hesitancy and their willingness to get vaccinated, nor the frequency of students’ outside activities. High-school students who experience negative moods during online studying should be monitored and receive additional support if the reopening is postponed. More opportunities to discuss COVID-19 prevention with family or friends, as well as a higher level of readiness, may increase the willingness to return to school on site. Local authorities and schools need to strengthen communication and coordination mechanisms to reduce parents’ and students’ schooling hesitancy by providing explicit information about the COVID-19 situation and risk-mitigation measures, along with normalizing messages about fear and anxiety.

## 1. Introduction

During the COVID-19 pandemic, schools were no longer a safe place. During the initial phase of the pandemic, the lockdown shifted the norm from on-site to online education. This was conducted to physically separate students and maintain a stable number of COVID-19 infections [1]. Even though evidence reported that children may not be potential COVID-19 spreaders [2,3], it may have been hard to make a decision about the school reopening because of the effects of the school reopening on disease spread [4], which depended on the prevalence of COVID-19 infection in each area [5,6], as well as its effects on stakeholders, including teachers, parents, and students [7]. Focusing on students during the pandemic, students were either a “risk to others” or “people at risk” [8]. As a “risk to others”, adolescents, unlike young children, had a more independent lifestyle and social life, including outside activities such as hanging out with peers, which may have resulted in a higher secondary-attack rate [9]. Changes in social activities and the rapid transition from on-site to online learning may have impacted students’ mental health as “people at risk” during unavoidable online classes [10]. Mental-health problems may have occurred if students were unable to adapt to online learning. In addition, adolescents were at risk of significant academic, social, and other health problems related to prolonged school closures.

Reopening schools as soon as possible not only provided benefits for students beyond educational outcomes but also increased the access to essential school-based services such as immunization, health, and psychosocial support. However, the transition from online to on-site education was difficult due to a lack of standardized school guidance for the school reopening. Even though schools have reopened, uncertainty about the COVID-19 situation and the capacity of the schools to maintain safe school operations to mitigate risks may have increased hesitancy among students and parents to participate in on-site studying. Aside from the high-school students’ knowledge, attitudes, and health-related behaviors regarding COVID-19 prevention reported in previous studies [11,12] and parents’ hesitancy [13], there is a lack of comprehensive evidence on student perspectives toward the transition to on-site studying. Decisions on reopening should require information on how schools, teachers, students, parents, and local communities respond to the school reopening and the pandemic situation. Rapid-response surveys of students and parents can provide information to stakeholders on how learning and well-being can best be supported during the educational transition in each context. Exploring factors related to students’ hesitancy to attend school on site and their well-being may provide supporting insights for a safe reopening [14].

To our best knowledge, there are no studies that investigated the factors associated with students’ hesitancy about on-site studying. The theoretical model of health beliefs and risk perception is an essential tool for understanding health and other behaviors. The Health-Belief Model (HBM) [15] is the most influential theory used to predict individual health behaviors and has been widely used for predicting behavior related to COVID-19-vaccine hesitancy in various populations [16,17,18]. Apart from sociodemographic factors, which are considered universal confounding factors, we hypothesized that individual beliefs based on the HBM may potentially be associated with schooling hesitancy among high-school students.

**Figure 1:**
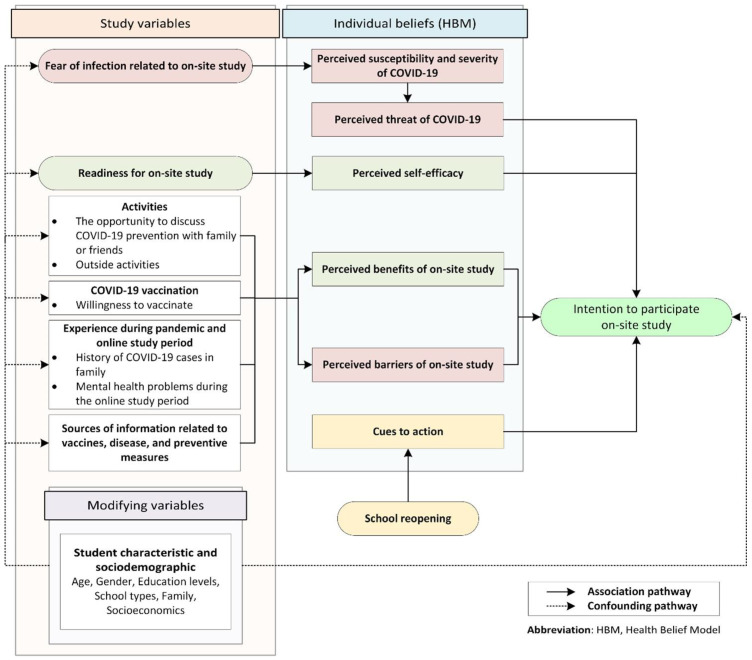
The conceptual framework of students’ hesitancy to participate in on-site studying.

According to the HBM, students’ hesitancy to participate in on-site studying could be influenced by a number of variables, including (1) perceived susceptibility, severity, and threat of COVID-19 (e.g., fear of COVID-19 infection related to on-site studying); (2) perceived benefits and perceived barriers of participating in on-site studying and factors that encourage or inhibit intention to participate in on-site studying (student’s experiences such as having experienced COVID-19 infection in family, being in a negative mood during online study; willingness to get vaccinated with the COVID-19 vaccine; student’s activities such as interactions with other people or family; or health-information seeking in the media or from healthcare personnel); (3) perceived self-efficacy (e.g., student’s readiness for on-site studying); and (4) cues to action (school reopening). The conceptual framework of the hypothesized factors related to students’ hesitancy to study on site is shown in Figure 1.

This study aimed to explore students’ hesitancy to attend school on site and the factors influencing student’s hesitancy. The primary focus was on high-school students, which may have had a greater impact on the educational situation during the pandemic. In addition, some student-related issues were also supplemented with parents’ perspectives. This study’s insights provide information for stakeholders to determine how to reduce students’ hesitancy to attend school on site and prevent negative consequences of the transition to regular education.

## 2. Materials and Methods

### 2.1. Study Design and Participants

**Figure 2:**
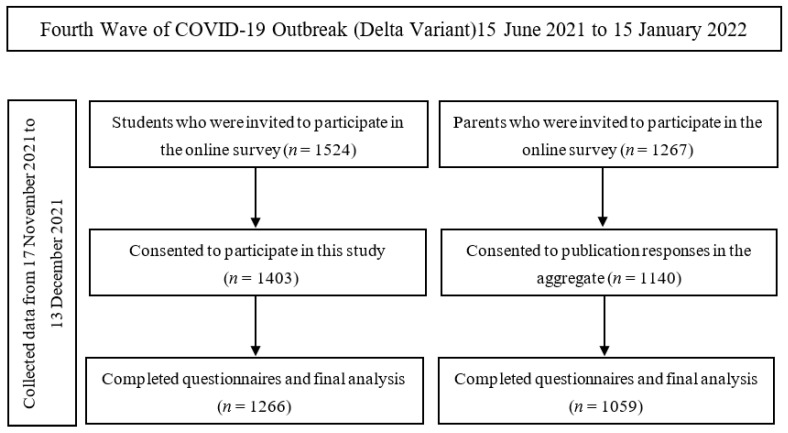
The study-flow diagram and pandemic period.

This cross-sectional study was conducted using an online survey from 17 November 2021 to 13 December 2021, near the end of the fourth wave of COVID-19 (Delta variant) in Thailand [19]. Survey data were collected and managed using REDCap (Research Electronic Data Capture), a secure, web-based software platform designed to facilitate data capture for research studies. An online survey was distributed to high-school students from all 26 schools located in an urban district of Chiang Mai (total population = 12,367). The study was publicized to encourage students to participate in this online survey via the school coordinators who were responsible for COVID-19 prevention in their schools. From a total of 1524 responses, 1403 participants consented to participate in the study. One thousand two hundred sixty-six participants (or 90.2%) completed all questions. Figure 2 shows a timeline of data collection and COVID-19 outbreak periods in Thailand. In addition, information from the parent section was gathered via an online survey distributed to the parents of students attending schools in the urban district of Chiang Mai via school coordinators.

### 2.2. Questionnaire Design

The students were asked about their behavior and activities in line with the self-assessment form of preventive measures for students in preparation for on-site studying announced by the Department of Health of Thailand and their emotions during on-site studying. The questionnaire about the perceptions of students and hesitancy to participate in on-site studying was designed in accordance with the adapted conceptual framework based on the HBM theory (Figure 1). The questionnaire consisted of four main parts:

(1) Personal information including socio-demographic characteristics (age, gender, grades, school type, and transportation to school), parents’ information (occupation, educational level, and income), and family information (primary parent, family members, elderly in family, and history of previous COVID-19 infections);
(2) Participants’ activities and behaviors during online classes, including the availability of alcohol-based hand sanitizer, the frequency and reason for going out for outside activities, the reasons for their opinion to be immunized, the use and disposal of masks, and their behaviors designed in accordance with the self-assessment form of preventive measures for students in preparation for on-site studying developed by the national committee, including public health experts and infectious-disease specialists from the Department of Health and educational experts from the Ministry of Education. Additionally, there was a question regarding their negative mood while taking online courses. A total of twenty questions could be answered using three categories: “never”, “sometimes”, and “always”;
(3) The perceptions of students regarding their readiness for on-site studying, their desire to be on site, and their fear of being infected if returning to classrooms on site. This part could be answered using five categories: “not at all”, “slightly”, “uncertain”, “very”, and “extremely”;
(4) The participants were asked about their sources of COVID-19 information, the most reliable sources, and their willingness to receive COVID-19 vaccines. The COVID-19-information sources included parents, teachers, friends, healthcare sector, television (TV), print media, foreign media, online media in Thailand, and social media. The question on willingness to receive COVID-19 vaccines could be answered using three categories: “Yes,” “Unsure,” and “No.”

For the purpose of supplementing parent assessment, self-reported questionnaires were used to collect parents’ sociodemographics, vaccination history, and decisions regarding their children’s vaccination and on-site education. Parents’ willingness to vaccinate their adolescent child with COVID-19 vaccine could be expressed using three categories: “Yes”, “Unsure”, and “No”. Parents’ willingness to allow their adolescent child to study on site could be expressed using three categories: “Not allowed to study on-site”, “Allowed to study on-site with online study”, and “Allowed to study on-site regularly”.

### 2.3. Statistical Analysis

All statistical analyses were performed using statistical software STATA (Stata Corp. 2019, Stata Statistical Software; Release 16; Stata Corp LLC, College Station, TX, USA). For the categorical data, personal information, details of outside activities, preventive or risky behaviors, willingness to get vaccinated, and COVID-19-information resources were described using a frequency and a percentage. The continuous variables were described using a mean with a standard deviation (SD) for parametric data or a median with an interquartile range (IQR) for non-parametric data. A comprehensive exploratory analysis using a multivariable logistic regression was conducted to identify factors associated with student’s hesitancy to study on site. The study findings were reported in accordance with the STROBE (Strengthening the Reporting of Observational Studies in Epidemiology) checklist. All statistical analyses were performed on a two-tailed basis, and a *p*-value of 0.05 was regarded as statistically significant.

### 2.4. Ethical Considerations

This study was conducted in accordance with the Declaration of Helsinki guidelines and the protocol was approved by the Research Ethics Committee, Faculty of Medicine, Chiang Mai University, Thailand (Study Code: COM-2564-08506). Informed consent was obtained from all subjects involved in the study.

## 3. Results

### 3.1. Characteristics of Participants

With a response rate of 10.2% of the total high-school students in the urban district’s schools, most of the 1266 students were female (64.0%) and attended private schools (83%). The mean age (±SD) of the participants was 16 years (±1). The majority studied in the tenth grade (46.0%), followed by the eleventh grade (37.9%). Almost half of the students had elderly family members in their household (45.7%). The highest proportion of family income was between 601 and 1200 USD/month (27.7%). Only 3.1% of students had COVID-19 cases in their family. The details of these characteristics are shown in Table 1.

### 3.2. Students’ Activities and Health-Related Personal Behaviors during Online-Study Period

**Figure 3:**
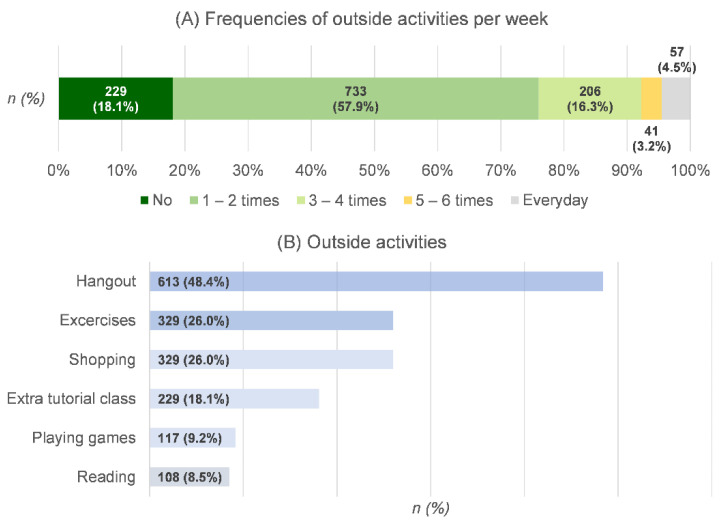
Students’ outside activities during the online-study period.

**Figure 4:**
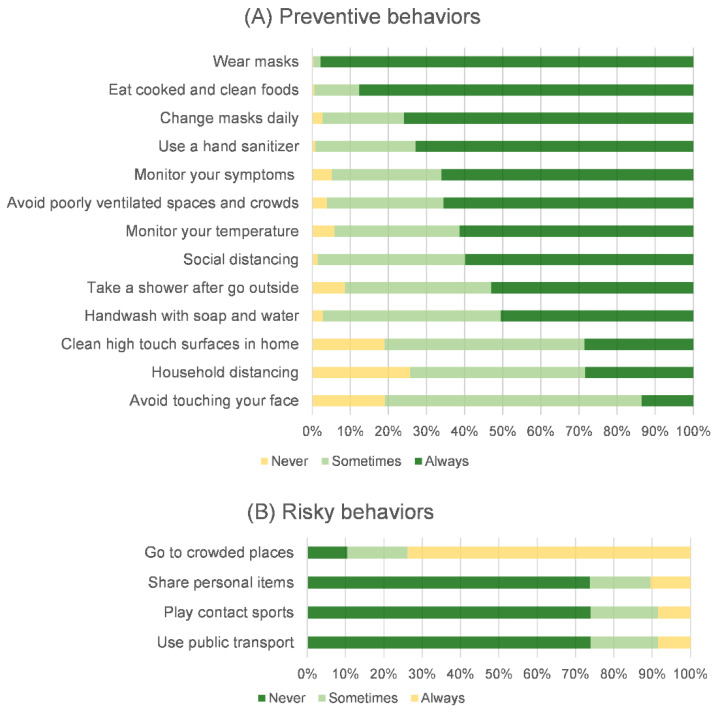
Preventive and risky behaviors of students.

Despite the absence of on-site classes in Chiang Mai during the COVID-19 pandemic, the majority of students (57.9%) engaged in outside activities from one to two times per week, whereas 18.1% did not participate in any outside activities. Only 4.5% of the students said they went outside every day. Regarding the details of outside activities, the students went outside to hang out as their most popular reason (48%). The next two most common reasons for going out were to get some exercise and to go shopping, each with a 26% share. Information about the outside activities of the students is shown in Figure 3. We also found that only 43.6% of the students had the opportunity to discuss COVID-19 prevention with family or friends during the online-study period. Regarding COVID-19-related behaviors, most students always performed preventive behaviors (more than 50%), except for cleaning high-touch surfaces at home, maintaining household distance, and avoiding touching their faces. Despite the 73.8% of the students reporting that they always went to crowded places, more than 90% of the students claimed to have never or sometimes engaged in other risky behaviors. This finding was particularly relevant to the frequency of outside activities during the absence of on-site classes. The details of the preventive and risky behaviors of students are shown in Figure 4.

### 3.3. Health-Information Receiving and Willingness to Get Vaccinated among Students

**Figure 5:**
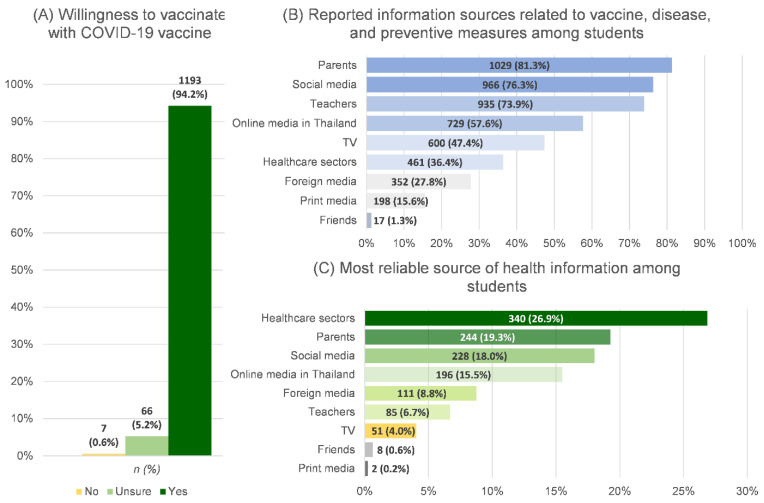
Students’ willingness to get vaccinated with COVID-19 vaccine and their health-information sources.

From the student perspective, the health sector (26.9%) was the most reliable source of COVID-19 information, followed by parents (19.3%) and social media (18.0%), while the top three sources from which the majority of students received information were their parents (81.3%), social media (76.3%), and teachers (73.9%). With 36.4%, the healthcare sector ranked sixth for information sources among the students. Most students (94.2%) responded that they were willing to receive the COVID-19 vaccination. The details of health-information receiving and willingness to get vaccinated among the students are presented in Figure 5.

### 3.4. Students’ Moods during the Online-Class Period and Their Attitude toward On-Site Studying

**Figure 6:**
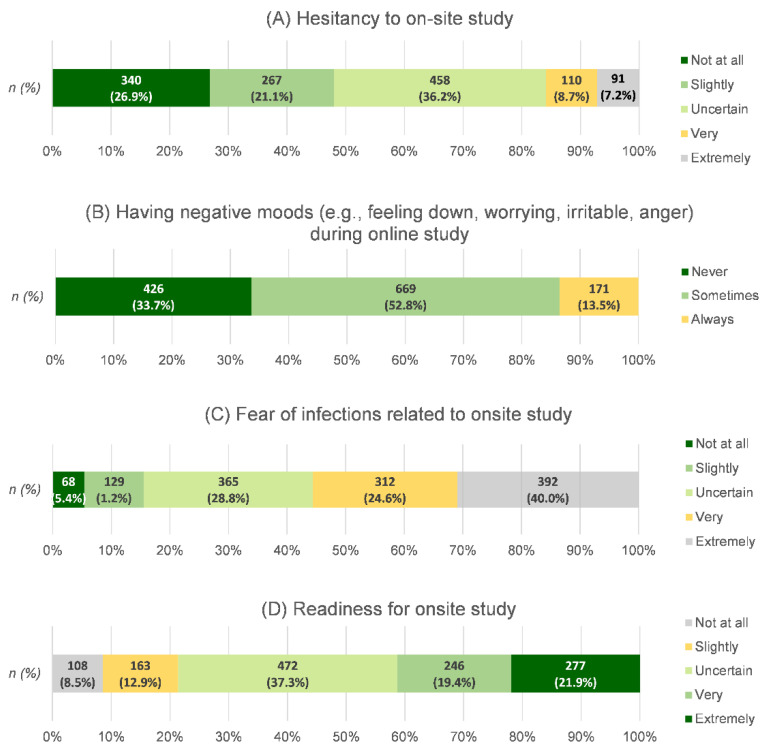
Students’ hesitancy, negative moods during online study, and attitudes toward on-site studying.

From Figure 6, almost half of the students (52.8%) said they were sometimes in a negative mood, and 13.5% said they were always in a negative mood. Concerns about COVID-19 infection related to on-site studying among the students showed a high proportion of extremely worried, 40%, followed by moderately worried, 28.8%, and very worried, 24.6%, respectively. High readiness levels (“very”/“extremely”) for on-site studying were reported in 41.3% of students, whereas 21.4% reported that they were not ready at all or were slightly ready for on-site education. Most of the students (52.1%) reported being hesitant to engage in on-site studying, ranging from uncertain to extremely hesitant. According to a survey of parents with adolescents in the same area, most participants (43.9%) did not allow their child to study on site, while only 17.6% allowed their child to regularly study on site (Figure S1A).

### 3.5. Factors Related to Students’ Hesitancy to Study on Site

A multivariable logistic regression was performed to explore the factors related to students’ hesitancy about on-site studying. As shown in Table 2, the students who were sometimes (aOR, 1.69; 95% CI, from 1.10 to 2.58; *p*, 0.016) or never (aOR, 1.93; 95% CI, from 1.22 to 3.03; *p*, 0.005) in a negative mood while studying online were significantly associated with a higher hesitancy about on-site studying, and this also applied to students who reported “very”–“extreme fear” of infection following on-site studying (aOR, 2.95; 95% CI, from 1.56 to 5.57; *p,* 0.001). The students who reported moderate (aOR, 0.28; 95% CI, from 0.14 to 0.58; *p*, 0.001) and “very”–“extremely” (aOR, 0.05; 95% CI, from 0.02 to 0.09; *p* < 0.001) degrees of readiness were significantly associated with lower odds of hesitancy about on-site studying. Having the opportunity to discuss with family or friends COVID-19 prevention (aOR, 0.71; 95% CI, from 0.54 to 0.94; *p*, 0.016) was also related to lower odds of hesitancy to be on site. No outside activities, sources of information related to vaccines, disease, preventive measures, or willingness to get vaccinated with COVID-19 vaccines were independently associated with students’ hesitancy.

## 4. Discussion

### 4.1. Key Findings: High-School Students’ Hesitancy to Attend School for On-Site Studying and Its Associated Factors

Approximately half of the high-school students in this study were hesitant to return to school. The majority of students (52.1%) reported being uncertain or extremely hesitant about engaging in on-site studying. In addition, 43.9% of parents with adolescents in the same area did not allow their children to study on site, which was higher than the percentage of parents who did not plan to send their children to school in person as reported by survey data (5%) from the US in 2021 [20] (Figure S1A,B). Our findings indicated that a less negative mood while studying online and a greater fear of infection after returning to school were factors significantly associated with increased on-site hesitancy. High levels of readiness and having the opportunity to discuss COVID-19 prevention with family or friends significantly reduced the odds of students’ hesitancy about on-site studying, while the frequency of outside activities was not a significantly associated factor.

### 4.2. Negative Moods during Online Study Related to Hesitancy about On-Site Studying

In this study, 13.5% of students reported being always in a negative mood during online classes, which is consistent with other studies on adverse mental health among Asian adolescents that reported percentages between 7.1% and 17% [21,22,23]. Adolescence, the transition between childhood and adulthood, was vulnerable to mental-health issues during the pandemic disruption due to maturity and physical, emotional, personality, and social development changes. A lack of interaction between students and their peers or teachers may also have affected their coping and mental health [24]. During online classes, it was difficult for students to ask for support from their teachers, which resulted in lower learning engagement [25]. Less negative moods during online education may indicate students’ adaptability and preference for this educational pattern. However, more than half of the students experienced negative moods during online-study time. Reopening schools provided more potential benefits for the majority. During transitional education, parents can support their children’s interest in learning by encouraging their curiosity and assisting them with online study [26]. In addition, students who consistently experience negative moods should be monitored and supported by the people surrounding them to enhance their well-being and implement appropriate learning activities if on-site studying is postponed due to an outbreak following the reopening [27,28,29].

### 4.3. Fear of Infection Causing Students’ Hesitancy to Study on Site

Nearly half of the students in this study exhibited a fear of being infected with COVID-19 that ranged from very high to extremely high, which was higher than that in previous studies in German adolescents (“worried to be personally infected” in 24.6% and “very worried to be personally infected” in 9.6%) [30] and Chinese adolescents (“Felt extreme fear” in 13.8%) [31] but comparable to the proportion in young adults in Chile (54.6% reported more fear since the pandemic) [32]. Students may be concerned about infection not only for themselves but also for their relatives [30]. Nearly half of the students had elderly relatives in the house, which may explain their fear of infecting their parents or vulnerable groups in their family. The different prevalence of fear of COVID-19 can be attributed to cultural contexts and other factors, such as differences in the access to medical care in different countries [33]. In the period during which the data were collected, COVID-19 vaccine for adolescents aged 12–17 years had recently been approved and was not generally available. Even though the majority of students in this study were willing to be vaccinated, the high extent of their fear of being infected could be explained by the aforementioned concerns. A greater fear of infection after returning to school had high independent effects on students’ hesitancy. To reduce students’ hesitancy, parents and teachers played an essential part in supporting students in order to reduce the fear of infection after returning to school on site [34,35]. Consistent performance in preventive management of the school and its employees could additionally enhance students’ and parents’ confidence in reopening schools [36].

### 4.4. Students’ Activities and Behaviors during Online Classes

The low frequency of outside activities per week, with more than half of students not going outside or only going outside 1–2 times per week, had no discernible impact on students’ hesitation. The most common outside activity among students was “hang out.” Since the context of the activity could be going to crowded places, this activity may additionally increase the risk of infection apart from the school reopening. However, it may increase students’ opportunities to discuss COVID-19 prevention with others, thereby decreasing the likelihood of students’ hesitancy. Exercise ranked second among the outside activities of students. However, this proportion represented only about half of the “hang out” activity. The decline in physical activities found in this study was consistent with that in previous studies and may have affected mental health in adolescents [37,38]. To encourage students to participate in physical activities after schools reopen, strong school policies and parental support were recommended [39,40].

According to global trends, adolescents consistently engage in most preventive behaviors and infrequently engage in risky behaviors [11,41,42]. Some behaviors, however, require to be more attentively addressed. Fewer than half of the students said they always cleaned high-touch surfaces, kept a safe distance from family members, and avoided touching their faces. Going to a crowded place was the only risky behavior they always did. In a different context, the same behavior led to different effective prevention strategies. More than half of students (60%) always kept a distance outside, while fewer kept a distance in their residences. Being among known family members may have led to lax preventive behavior, potentially increasing the risk of home transmission [43]. To prevent the outbreak following the school reopening, the students and the people surrounding them, including their parents and teachers, should be continually engaged in preventive measures and made aware of the potential risk posed by their behaviors.

### 4.5. Student Readiness to Attend School on Site

Nearly 40% of students reported that they were very–extremely ready to return to school on site, while the same percentage reported uncertainty. Even though students’ readiness to attend school on site was uncertain, it was also shown to be positively associated with less student hesitancy about on-site studying. However, there were no reported data on students’ on-site studying readiness prior to attending school on site. Strengthening students’ skills and creating a learning-friendly environment, along with sufficient social support, could enhance their school readiness [29]. Both parents’ support and schools’ well-preparedness are needed for a smooth educational transition.

### 4.6. Source of Information and Willingness to Get Vaccinated

According to students’ reports, the health sector, parents, and social media were the topmost reliable sources of COVID-19 information. Our study found that parents remained important, accessible, and reliable sources of health information for students, in accordance with a previous study [44]. Aside from the high willingness to get vaccinated among the students in our study, 78.1% of parents in the same area (Chiang Mai’s urban district) agreed that their children should be vaccinated (Figure S1C). These findings were consistent with previous research findings relative to either adolescents or parents [45,46] (Figure S1C) [20]. Immunization may not be as frightening for adolescents as it is for younger children, whose parents were less likely to allow them to be vaccinated [47]. Although vaccination willingness was not significantly associated with students’ hesitancy to study on site, vaccination coverage among students remains crucial and must be addressed to decrease the fear of infection and disease transmission in the community.

### 4.7. Implications

According to the findings of this study, people around students, including parents and teachers, can empower students’ willingness to return to school for on-site studying. In addition, it was important to strengthen students’ readiness to go on site, encourage them to discuss COVID-19-prevention issues with family or friends, and reduce their fear of infection. In comparison with other studies on schooling hesitancy [7,40], this study supported that students’ belief and their surroundings remained important determinants. Regarding the consequences of online studying, students’ mental health could help to identify those unsuited for online learning who need continuous monitoring and additional support if the reopening is postponed. Lowering students’ and parents’ hesitancy about on-site studying should be emphasized to prevent negative consequences and educational inequity as a result of being unable to access the academic, social, emotional, and other benefits of regular education. Local authorities and schools need to strengthen communication and coordination mechanisms that reduce parents’ and students’ schooling hesitancy by providing clear, concise, and accurate information about the COVID-19 situation, guidance for disease prevention in schools, protocols for infected students after the reopening, and normalizing messages about fear and anxiety. According to the high percentage of those willing to get vaccinated, stakeholders and public health sectors should collaborate to increase vaccine coverage among high-school students, which could reduce the fear of infection and promote intention to participate in on-site studying. These actions are required to reassure parents and students that schools are safe for on-site studying.

### 4.8. Strengths, Limitations, and Generalizability

The strength of this study was in providing comprehensive information that may influence the transition from online to on-site education, including students’ physical activities, consistent preventive measures, and students’ well-being. Online study’s consequences on students’ mood, readiness, and hesitancy about returning to school on site supported the controversial gap between practices to prevent COVID-19 transmission and school reopening [48]. This study still had several limitations. First, the causal associations could not be determined due to the limitations of cross-sectional studies. Hence, the relationships among activities, readiness, and students’ willingness to study on site should be interpreted carefully. However, in order to provide timely information to stakeholders, a cross-sectional study is the most effective epidemiological design and can be used to explore the factors associated with schooling hesitancy among high-school students in a particular period (e.g., prior to reopening, part of the reopening process, or after reopening). Second, because the majority of the students in this study attended private schools, generalizability should be approached with caution due to representative biases. Third, the study data were collected from November to December 2021, while the Delta strain of COVID-19 was decreasing in Thailand [19]; because the data were specific to the Thai setting prior to the Omicron-variant pandemic, generalization to any other context may not applicable and should be approached with caution.

Further research on the association between schooling hesitancy and mental health among high-school students is suggested to clarify more specific mental-health problems utilizing standardized screening instruments. A longitudinal study during the transition from online to new-normal education is suggested to represent the dynamics of changes among students and educational systems. Besides high-school populations, future research should investigate younger age groups of students, who may have different sets of influencing factors, since schooling hesitancy in these populations is mainly influenced by parental attitude and belief and many studies showed higher parental hesitation related to on-site studying, vaccination, and other child health behaviors.

## 5. Conclusions

High-school students who experienced negative moods during the online-study period should be monitored and receive additional support if the reopening is postponed. More opportunities to discuss COVID-19 prevention with family or friends, as well as a higher level of readiness, may increase the willingness to return to school on site. Local authorities and schools need to strengthen the communication and coordination mechanisms to reduce parents’ and students’ schooling hesitancy by providing explicit information about the COVID-19 situation, disease prevention in schools, and protocols for infected students after the reopening, along with normalizing messages about fear and anxiety. These actions are required to reassure parents and students that schools are safe for on-site studying to prevent educational inequity and prevent negative consequences after the reopening.

## Figures and Tables

**Table 1:** Students’ characteristics and sociodemographics.

Characteristics		(N = 1266)	
		*n*	%
Age (year)	Mean ±SD	16	±1
Gender	Male	456	36.0
	Female	810	64.0
Education levels	10th grade	582	46.0
	11th grade	480	37.9
	12th grade	204	16.1
School types	Private	1050	83.0
	Government	103	8.1
	University demonstration	113	8.9
Living with	Parents	1203	95.0
	Relatives	63	5.0
Number of family members	Less than three	133	10.5
	From three to five	935	73.9
	More than five	198	15.6
Elderly members	Yes	578	45.7
	No	688	54.3
History of COVID-19 cases in family	Yes	39	3.1
	No	1227	96.9
Family income (USD per month)	≤600	181	14.3
	601–1200	351	27.7
	1201–1800	275	21.7
	1801–2400	180	14.2
	2401–3000	121	9.6
	>3000	158	12.5

**Table 2:** Factors related to on-site-study hesitancy of high-school students.

Factors		aOR	95%CI	*p*-Value
Students’ Attitudes				
Being in a negative mood during the online-study period	Always (13.5%)	(ref.)		
	Sometime (52.8%)	1.69	1.10–2.58	0.016
	Never (33.7%)	1.93	1.22–3.03	0.005
Readiness for on-site studying	Not at all/slightly (21.4%)	(ref.)		
	Uncertain (37.3%)	0.28	0.14–0.58	0.001
	Very/Extremely (41.3%)	0.05	0.02–0.09	<0.001
Fear of infection related to on-site studying	Not at all/slightly (64.6%)	(ref.)		
	Uncertain (24.6%)	1.65	0.85–3.19	0.137
	Very/Extremely (6.6%)	2.95	1.56–5.57	0.001
**Activities**				
Having the opportunity to discuss COVID-19 prevention with family or friends.	(43.6%)	0.71	0.54–0.94	0.016
No outside activities	(18.1%)	1.36	0.96–1.92	0.079
**Student’s sources of information related to vaccines, disease, and preventive measures** **(Received vs. Not received (ref.))**				
Parents	(81.3%)	1.07	0.72–1.57	0.747
Teachers	(76.3%)	0.87	0.62–1.21	0.407
TV	(73.9%)	0.89	0.66–1.18	0.410
Print media	(57.6%)	1.15	0.77–1.73	0.495
Online media in Thailand	(47.4%)	0.95	0.72–1.26	0.722
Healthcare sectors	(36.4%)	1.22	0.91–1.65	0.179
Social media	(27.8%)	0.77	0.55–1.07	0.117
Foreign media	(15.6%)	0.82	0.59–1.13	0.224
Friends	(1.3%)	1.24	0.39–3.92	0.716
Willingness to get vaccinated with COVID-19 vaccines	No (0.6%)	(ref.)		
	Unsure (5.2%)	0.83	0.11–6.25	0.861
	Yes (94.2%)	0.74	0.11–5.13	0.759

Abbreviations: aOR, adjusted odds ratio; ref., reference category. The magnitude of associations (aOR) were obtained with an exploratory analysis using a multivariable logistic regression with adjustment for student’s socio-demographics including gender, study grades, school types, family income, number of family members, and history of COVID-19 cases in family.

## Data Availability

The data presented in this study are available upon request from the correspondent author.

## Supplementary Materials

The following supporting information can be downloaded at: https://www.mdpi.com/article/10.3390/ijerph19159261/s1, Figure S1: Parents’ willingness to vaccinate their children and hesitancy to allow their children to study on site.
