# Supplementary material for: Focus on High School: Factors Associated with Creating Harmony between the Educational Transition and Adolescents’ Well-Being
Source: Int J Environ Res Public Health. 2022 Jul 28;19(15):9261. doi: 10.3390/ijerph19159261 (PMC9368253; doi:10.3390/ijerph19159261)
Supplement: Supplementary file 1 [file ijerph-19-09261-s001.zip › ijerph-1807263-supplementary.pdf]

Figure S1: Parents' willingness to vaccinate their children and hesitancy to allow their children to study on site.

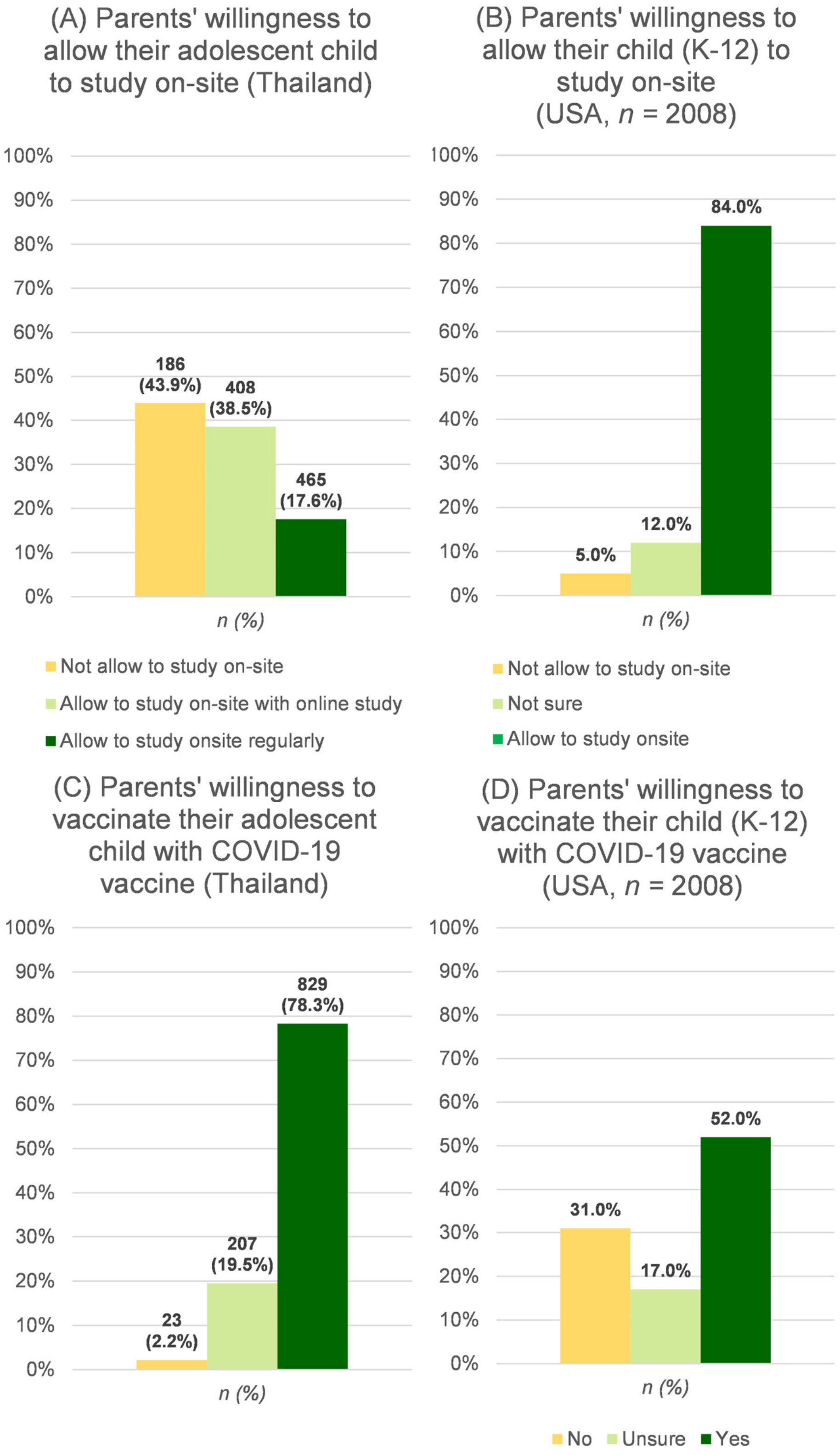

Comparative data from Schwartz HL, et al. Will Students Come Back? School Hesitancy Among Parents and Their Preferences for COVID-19 Safety Practices in Schools. Santa Monica, CA: RAND Corporation, 2021.
